# Navigating Food Allergies: Advances in Diagnosis and Treatment Strategies

**DOI:** 10.7759/cureus.56823

**Published:** 2024-03-24

**Authors:** Nikhil Chowdary Peddi, Sudheer Kumar Muppalla, Himabindu Sreenivasulu, Sravya Vuppalapati, Myna Komuravelli, Rahul Navab

**Affiliations:** 1 Pediatrics, St. Barnabas Hospital Health System, New York, USA; 2 Pediatrics, PES Institute of Medical Sciences and Research, Kuppam, IND; 3 General Physician, PES Institute of Medical Sciences and Research, Kuppam, IND; 4 Pediatrics, Chalmeda Anand Rao Institute of Medical Sciences, Hanamkonda, IND; 5 Internal Medicine, PES Institute of Medical Sciences and Research, Kuppam, IND

**Keywords:** non-ige mediated, ige-mediated hypersensitivity, omalizumab, food allergy quality of life, food and nutrition

## Abstract

Food allergy is a major health concern worldwide, encompassing both immunologic and non-immunologic reactions. This review thoroughly examines the pathophysiology, clinical manifestations, and treatment options for various types of food allergies. Immunologic food allergies, including IgE-mediated reactions such as oral allergy syndrome and systemic anaphylaxis, pose various diagnostic and management challenges. Non-IgE-mediated reactions such as food protein-induced enterocolitis syndrome, dermatitis herpetiformis, and proctocolitis necessitate individualized patient care. In addition, mixed reactions such as eosinophilic esophagitis and atopic dermatitis complicate the clinical picture. Skin prick tests, serum-specific IgE tests, and oral food challenges are all necessary for accurate food allergy diagnosis. The primary therapeutic options are allergen avoidance, epinephrine-based emergency management, and emerging treatments like immunotherapy. Our review emphasizes the importance of multidisciplinary collaboration and ongoing research in improving our understanding and managing food allergies, promising a brighter future for those affected.

## Introduction and background

Food allergy is considered one of the major problems we deal with in today's modern world. It is considered to be a part of one of the four symptoms of atopic March, along with asthma, eczema, and allergic rhinitis. A food allergy can be categorized as immunoglobulin E-mediated, non-immunoglobulin E-mediated, or mixed, depending on the pathophysiologic immunological processes underlying it. Food allergy sufferers and those who care for them bear great psychological, social, emotional, and financial costs. There is also a significant impact on the healthcare system. Pediatricians frequently deal with these patients as primary healthcare professionals and are called upon to identify and treat food allergies [1].

## Review

Food allergy: pathophysiology and types

Food allergy can be further classified into two categories based on the underlying pathophysiology. The first category is immunologic food allergies, which are primarily driven by IgE-mediated reactions, which include oral allergy syndrome and systemic anaphylaxis, and non-IgE-mediated reactions, which include food protein-induced enterocolitis syndrome (FPIES), dermatitis herpetiformis, and proctocolitis [2]. The second category is non-immunologic, which includes lactose intolerance [2].

Food allergy was defined by an expert panel of the National Institute of Allergy and Infectious Diseases as "an adverse health effect arising from a specific immune response that occurs reproducibly on exposure to a given food [3]." Basically, all immune-mediated responses, including those brought on by the innate and adaptive immune systems, are included in this response [4]. IgE attaches to mast cells and basophils via the high-affinity receptor Fc region of IgE (FcεRI) after interacting with dietary antigens, forming a cross-linked state; when these cells are activated, granules containing already formed inflammatory mediators (like histamine) are released. Additionally, inflammatory cytokines (like IL-4), chemotactic molecules, proteases (like tryptase), and inflammatory mediators (like leukotrienes) can be synthesized a new or released. An acute allergic reaction occurs when symptoms appear soon after allergen exposure as mast cells and basophils are triggered within minutes of IgE cross-linking [5].

Food allergies can cause symptoms to appear directly at the sites of contact with the allergens (such as the mouth, esophagus, and/or intestine) or in other organs because they reach the bloodstream through the gastrointestinal system. When allergens that may cross-link IgE attached to effector cells breach the mucosal barrier and enter the bloodstream, systemic responses occur. Reactions to allergens can also impact the neurological and circulatory systems [5].

The amount of ingested allergen, its stability against digestion, and the permeability of the epithelial barrier are factors that affect the kind and intensity of responses. An acute allergic reaction might cause severe inflammation that may be fatal. Anaphylactic shock and vascular collapse may result from the circulation's release of vasoactive mediators [6].

Immunologic: IgE mediated 

IgE-mediated food allergies may cause severe reactions and, in certain cases, lead to hospitalizations and severe psychological distress. It is important to know the clinical presentation in order to deal with them effectively.

Oral allergy syndrome (OAS)

Oral allergy syndrome (OAS) is a disorder marked by IgE-mediated immediate allergic symptoms limited to the oral mucosa. These symptoms can include sudden onset vascular edema of the lips, tongue, palate, and pharynx, itching, stinging pain, and ear itching. Sometimes, the condition is accompanied by a tightness in the throat. These symptoms usually go away with time. Oral mucosal symptoms, which arise when a patient with birch pollen allergy eats a food belonging to the Rosaceae family (apple, cherry, peach, etc.), are a common example of OAS [7].

For example, food allergens that cause OAS easily dissolve in the mouth and are broken down by digestive enzymes found in gastric juice. The term "class 2 food allergy" was coined to distinguish food allergies caused by cross-reactivity between proteins in fruits and vegetables and pollen antigens in individuals sensitized by the antigen via the airway. This was because the properties of these food allergens differed from those of known food allergens that are resistant to digestive enzymes and induce sensitization via the intestine (class 1 food allergy) [8].

One common illness that displays OAS is pollen food allergy syndrome (PFS), as was previously discussed. Apart from PFS, another condition that has been documented to demonstrate OAS is latex-fruit syndrome (LFS), which is an allergy to fresh fruits or vegetables following sensitization with latex-inhalation antigen in latex powder [7]. Clinically, some patients whose skin test results are positive also report mouth pain just after eating an egg during an oral challenge test, but if they keep eating the egg, they do not exhibit systemic allergy symptoms [7].

Therapeutic options for OAS: Therapy for food allergies varies greatly due to the multiplicity of criteria for the disorder and divergent views on what exactly qualifies as pervasive food additives. According to 2003 research by Ma et al. of US allergists, Pollen Food Allergy Syndrome (PFAS) is widely misclassified as being identified in individuals with class 1 food allergies [9]. This might result in inadequate management of food allergies that could be fatal, though, depending on how those allergists handle PFAS [10].

In a research conducted in the United States, 70% of allergists stated that they would use an epinephrine auto-injector (EAI) for treating PFAS/OAS, but just 18% of allergists in the United Kingdom had never suggested using one [9,11]. In this study, US allergists reported that they required an EAI due to a response involving symptoms related to the lower airways (89%), pharyngeal symptoms (58%), face angioedema (31%), and generalized urticaria (19%) [11].

Apart from avoidance, education, or EAI, research has looked into subcutaneous immunotherapy (SCIT) and sublingual immunotherapy (SLIT) to examine if tolerance to provocative foods may be developed, and the research did not show much significance. This is because pollen is usually the predominant sensitizer in class II allergens [12].

Systemic anaphylaxis

An acute, potentially fatal systemic hypersensitivity response called anaphylaxis is often thought to be an immunoglobulin (Ig) E-mediated reaction; however, immunologic events associated with immune complex complement or IgG can also result in anaphylaxis by causing mast cell degranulation. Food allergies, followed by medications, most frequently cause anaphylaxis. Anaphylaxis patients typically exhibit cutaneous or mucous membrane symptoms, followed by respiratory and gastrointestinal problems [13].

Any one of the following conditions described will meet the criteria of anaphylaxis: The rapid development of a disease affects the skin, mucosal tissue, or both (e.g., widespread hives, pruritus or flushing, swelling lips, tongue, or uvula), as well as one or more of the following symptoms which include breathing difficulties (such as wheezing, bronchospasm, stridor, decreased peak expiratory flow, and hypoxemia), or lower blood pressure or related end-organ dysfunction symptoms (such as incontinence, syncope, or hypotonia (collapse)) [14].

Two or more of the following that happen quickly, from a few minutes to many hours, following the patient's likely exposure to an allergen which includes the involvement of the mucosal tissue or the skin (e.g., flushing, pruritus, or swelling lips, tongue, or uvula); respiratory compromise, including hypoxemia, bronchospasm, stridor, dyspnea, and wheezing; decreased blood pressure or related symptoms, such as incontinence, syncope, or hypotonia (collapse); or chronic gastrointestinal problems (vomiting, cramping in the abdomen, etc.) [14].

Blood pressure is dropped (i.e., minutes to many hours) following exposure to a known allergen for the patient [14]. In kids, low systolic blood pressure (age-specific) or a drop in systolic blood pressure of at least 30% and for adults a drop in baseline blood pressure of 30% or more or systolic blood pressure of less than 90 mm Hg [14].

Therapeutic options for systemic anaphylaxis: Adrenaline is the first-line treatment for anaphylaxis and save lives; early detection and rapid administration of the medication are essential to the acute care and treatment of the condition [15]. An epinephrine auto-injector prescription can be written for any of the following six absolute indications: prior idiopathic anaphylaxis (IA); prior exercise-induced anaphylaxis; prior venom allergy in adults with prior systemic responses (unless receiving maintenance venom immunotherapy (VIT)) and in children with more than systemic cutaneous reactions; previous underlying mast cell dysfunction and any prior systemic reaction; and unstable or moderate to severe, chronic asthma with food allergy [16].

First-line therapies for anaphylaxis do not include corticosteroids or antihistamines. According to the guidelines, antibiotics and corticosteroids should only be administered in addition to epinephrine [17-19]. Patients with reactive airway disease or any patient exhibiting indications of an active bronchospasm are treated with beta2 agonists. Since glucagon possesses inotropic and chronotropic actions not mediated by beta receptors, it should be administered to patients with resistant hypotension, including those receiving beta-blocker treatment [17,19,20].

Non-IgE mediated 

Food allergies, encompassing both IgE- and non-IgE-mediated reactions, pose significant challenges in diagnosis and management [21]. Non-IgE-mediated food allergies specifically examined food protein-induced enterocolitis syndrome (FPIES), dermatitis herpetiformis, and proctocolitis. Understanding the distinct clinical presentations and therapeutic strategies for these conditions is crucial for effective patient care.

Food Protein-Induced Enterocolitis Syndrome (FPIES)

FPIES is a non-IgE-mediated gastrointestinal food hypersensitivity, often affecting infants [22]. Common triggers include cow's milk and soy. Symptoms include severe vomiting, diarrhea, and lethargy, leading to dehydration and potentially life-threatening complications.

Therapeutic options for FPIES: The cornerstone of FPIES management involves eliminating the triggering food(s) from the diet [23]. Extensive hydrolyzed formulas may be recommended for infants, while older children may require strict avoidance. Gradual reintroduction of the trigger food under medical supervision helps assess tolerance [21]. This is crucial for expanding the limited diet of FPIES patients.

Proactive management of reactions: In severe cases where accidental exposures can occur, having a written emergency plan and administering fluid resuscitation, if needed, are vital components of FPIES management [21].

Dermatitis Herpetiformis

Dermatitis herpetiformis is a chronic, blistering skin condition associated with gluten sensitivity [24]. It is a manifestation of gluten-sensitive enteropathy and shares genetic and immunologic features with celiac disease.

Therapeutic options for dermatitis herpetiformis: The primary treatment for dermatitis herpetiformis involves strict adherence to a gluten-free diet [25]. Eliminating gluten-containing grains can resolve skin lesions. Dapsone, an anti-inflammatory medication, is often used to manage symptoms and skin lesions [26]. Regular monitoring for potential side effects, including blood disorders, is essential. Topical or systemic corticosteroids may be prescribed for acute flares, providing symptomatic relief while awaiting the benefits of a gluten-free diet [22].

Proctocolitis

Proctocolitis, often seen in infants, is characterized by inflammation of the rectum and colon [27]. It is seen with consuming specific food antigens, with cow's milk being a common culprit.

Therapeutic options for proctocolitis: The first-line approach eliminates the offending food(s) from the infant's or breastfeeding mother's diet [28]. A switch to hypoallergenic or extensively hydrolyzed formulas may be considered for formula-fed infants.

Exclusive breastfeeding for at least six months is recommended for breastfeeding infants [29]. If the mother is breastfeeding, she may need to eliminate specific allergens from her diet. Regularly monitoring symptoms and systematically reintroducing the eliminated foods can help identify the causative allergen and guide ongoing management [23].

In conclusion, the diverse spectrum of non-IgE-mediated food allergies, including FPIES, dermatitis herpetiformis, and proctocolitis, requires tailored diagnostic and therapeutic approaches [21,24,27]. Advancements in our understanding of these conditions and ongoing research efforts contribute to developing effective management strategies, emphasizing the importance of multidisciplinary collaboration in providing optimal care for affected individuals.

Mixed IgE and non-IgE

Eosinophilic Esophagitis (EoE)

Eosinophilic esophagitis (EoE) typically manifests as dysphagia or food impactions in older children and adults, but other symptoms may occur. Feeding dysfunction, failure to thrive, abdominal pain, nausea, and vomiting are manifestations that are particularly severe in young children [30]. Diagnosing EoE requires identifying ≥15 eosinophils per high-power field on esophageal biopsy via upper endoscopy and esophageal dysfunction symptoms [31]. EoE is generally considered a chronic disease, and if left untreated, it can result in esophageal remodeling [32]. This remodeling can cause decreased esophageal distensibility and the formation of strictures.

Therapeutic options for EoE: The mainstays of treatment are proton pump inhibitors (PPIs), preparations of swallowed steroids, and/or avoidance of select trigger foods. Most patients will respond to one or all of these approaches [33].

Atopic Dermatitis

Atopic dermatitis is children's most common inflammatory skin disorder, accounting for up to 30% in industrialized countries. It is characterized by recurrent episodes of dry skin, pruritus, and frequent skin infections. Chronic pruritus, lack of sleep, limitations in social activities, and significant loss of productivity at work all hurt the quality of life for patients and caregivers [34]. Atopic dermatitis increases the risk of food sensitization and IgE-mediated food allergies [35].

Therapeutic options for atopic dermatitis: Emollients and topical anti-inflammatory medications treat atopic dermatitis by reducing inflammation and repairing skin barrier defects [36].

High-risk infants should be exclusively breastfed until four months, while hydrolyzed formula is recommended for those who cannot breastfeed. However, there is limited evidence to support the prevention of food allergies and atopic dermatitis in high-risk breastfeeding patients. According to some studies, breastfeeding may prevent cow's milk allergy by age two [37]. Further research into the pathophysiology of atopic dermatitis and its impact on food allergy development can lead to more effective therapeutic targets for prevention and treatment.

Diagnostic approach

A complete blood test is required to accurately diagnose allergies against a specific range of food items. The clinical history and examination are the first-line methods for determining food allergy. In general, food allergy diagnostic tests (e.g., skin prick test (SPT), serum-specific IgE tests, and oral food challenges) should be performed by an allergist [38]. Responsible food pricks into the skin of a positive SPT causes a wheal and flare response. Its specificity is just about 50%, whereas its sensitivity is over 90%. Therefore, a positive SPT by itself cannot indicate a food allergy; the patient also needs a positive medical history [38].

To reduce the number of false positives, over-testing with SPT is avoided. SPT should only be performed on foods relevant to the patient's history. A negative SPT has a negative predictive value greater than 95%, indicating the absence of IgE-mediated reactions [39,40]. While serum-specific IgE tests are less sensitive and more expensive than SPTs, they can still diagnose food allergies, especially if SPTs are not possible or unavailable [41].

Oral food challenges involve gradually feeding the suspected food while carefully monitoring for any symptoms under medical supervision. In the event of symptoms, feeding is stopped, and the patient is treated as needed. Food challenges should only be held in clinics or hospitals with the personnel and equipment to treat anaphylaxis [42].

The basophil activation test (BAT), which evaluates in vitro basophilic activation by specific allergens, is another test for detecting sensitization. A recent study found that BAT effectively distinguishes between allergy and tolerance in peanut-sensitive children, with 97% accuracy, 95% positive predictive value, and 98% negative predictive value [43]. Epigenetic markers, particularly DNA methylation, have diagnostic value for atopic sensitization, asthma, allergic rhinitis, and food allergy and can predict clinical responses in controlled allergen challenges, such as oral food challenges [44].

Another strategy for diagnosing food allergies is to follow an elimination diet and keep food/symptom diaries. The elimination diet can help diagnose and treat food allergies. It entails completely avoiding suspected foods or food groups for a set period (typically one to two weeks) while monitoring decreased symptoms [39,40]. If a food allergy is suspected, the food is avoided, an epinephrine auto-injector is prescribed, and the patient was referred for allergy testing [38].

Therapeutic approach

The only way to treat food allergies is to stay away from the foods that trigger them. The offending allergen has to be eliminated from the diet as soon as a food allergy has been established [38]. The identified food allergens are eliminated from the diet permanently when the elimination diet is used as a therapy unless there is evidence that the food allergy has been cured [39,45].

Intramuscular epinephrine in the lateral thigh is the preferred treatment for accidental exposure [39,45]. In North America, two epinephrine auto-injectors are available: the EpiPen® and Twinject®. Both products come in two dosages (0.15 mg and 0.30 mg), prescribed based on body weight. The 0.30 mg dosage should be used for people weighing 30 kg or more, while the 0.15 mg dosage should be used for children weighing 15-30 kg. Certain sources recommend switching to a 0.30 mg dose at 25 kg rather than 30 [46].

Every person receiving emergency epinephrine has to be taken right away to a hospital for assessment and monitoring (ideally by ambulance) [14]. Additionally, patients and their carers need to be taught how to avoid certain foods, identify and handle allergic and anaphylactic responses, safely use epinephrine auto-injectors, and seek emergency medical help [38]. Individuals should also be taught to read food labels carefully, looking for hidden ingredients like "natural flavor" or "spices" that could indicate the presence of allergens, as well as "may contain" warnings. All food allergy patients should obtain medical identification [38].

Additionally, recent studies have demonstrated that children with peanut allergies might get desensitized to peanuts by progressively consuming more under careful supervision [47]. Analogously, studies on allergy to milk and eggs have been done. Although adverse effects have been recorded, methods are still being established, and the research only included a limited number of subjects; the findings are encouraging. Therefore, a more confirmatory study in this field is required [38].

Omalizumab

A recent study found that omalizumab may help people with IgE-mediated food allergies consume a wider range of foods and increase their food dosage. As an oral immunotherapy (OIT) adjuvant, omalizumab (OMA) can help with higher maintenance doses and high-dose desensitization [48,49]. Omalizumab (OMA), an anti-IgE monoclonal antibody (mAB), has been studied in individuals with IgE-mediated food allergy (FA) both as monotherapy and as an adjuvant to OIT (OMA+OIT) in order to address the need for a treatment that goes beyond allergen avoidance [50,51]. OMA binds to IgE antibodies and blocks them from interacting with basophil and mast cell FcεRI receptors, which inhibits degranulation and the release of allergy response mediators [50,51]. Nasal polyposis, chronic spontaneous urticaria, and moderate-to-severe allergic asthma are among the indications for which OMA has been given approval [52]. OMA may have therapeutic use in the management of IgE-mediated FA due to its mechanism [53,54].

Using OMA+OIT not only aids in the creation of physiologically inactive anti-IgE-antibody complexes but also downregulates FcεRI expression and causes immunological repolarization toward T-helper 1 cells. This suggests that OMA efficiently inhibits free IgE molecules. Thus, via downregulating FcεRI expression in basophils, decreasing allergen-specific IgE levels, and raising IgG4 levels, the combination of OMA+OIT can result in allergen desensitization [49]. Recently, in 2024, OMA has been approved by the FDA as a treatment for food allergies among children >1 year of age and adults which can help mitigate the harmful reactions from food allergies without restricting or limiting their diets so they can still enjoy the nutritious values of these foods [55]. Given these new findings, it is reasonable to assume that the future of food allergy management is promising and will help many people who suffer from multiple food allergies, but further studies and research are always warranted to help with the well-being of patients with food allergies.

## Conclusions

Food allergies pose complex challenges, affecting people physically, emotionally, and financially. Effective management necessitates a thorough understanding of the wide range of immunologic and non-immunologic reactions and personalized diagnostic and therapeutic strategies. While current treatments primarily concern allergen avoidance and emergency interventions, emerging therapies such as immunotherapy provide hope for better outcomes. Continued research and multidisciplinary collaboration are critical to advancing our understanding and management of food allergies, ultimately improving the quality of life for those affected and paving the way for novel approaches to prevention and treatment.
